# REstricted Fluid REsuscitation in Sepsis-associated Hypotension (REFRESH): study protocol for a pilot randomised controlled trial

**DOI:** 10.1186/s13063-017-2137-7

**Published:** 2017-08-29

**Authors:** Stephen P. J. Macdonald, David McD Taylor, Gerben Keijzers, Glenn Arendts, Daniel M. Fatovich, Frances B. Kinnear, Simon G. A. Brown, Rinaldo Bellomo, Sally Burrows, John F. Fraser, Edward Litton, Juan Carlos Ascencio-Lane, Matthew Anstey, David McCutcheon, Lisa Smart, Ioana Vlad, James Winearls, Bradley Wibrow

**Affiliations:** 1 Centre for Clinical Research in Emergency Medicine, Harry Perkins Institute of Medical Research, Perth, WA Australia; 2 Emergency Department, Royal Perth Hospital, Perth, WA Australia; 3 Emergency Department, Austin Hospital, Melbourne, VIC Australia; 4 Department of Medicine, University of Melbourne, Melbourne, VIC Australia; 5 Emergency Department, Gold Coast University Hospital, Gold Coast, QLD Australia; 6 School of Medicine, Bond University, Gold Coast, QLD Australia; 7 School of Medical Sciences, Griffith University, Gold Coast, QLD Australia; 8 Emergency Department, Fiona Stanley Hospital, Perth, WA Australia; 9 Emergency and Children’s Services, The Prince Charles Hospital, Brisbane, QLD Australia; 10 Emergency Department, Royal Hobart Hospital, Hobart, TAS Australia; 11 Division of Emergency Medicine, Medical School, University of Western Australia, Perth, WA Australia; 12 Department of Intensive Care, Austin Hospital, Melbourne, VIC Australia; 13 School of Medicine, University of Melbourne, Melbourne, VIC Australia; 14 School of Medicine and Pharmacology, University of Western Australia, Perth, WA Australia; 15 Critical Care Research Group, The Prince Charles Hospital, Brisbane, QLD Australia; 16 School of Medicine, University of Queensland, Brisbane, QLD Australia; 17 Department of Intensive Care, Fiona Stanley Hospital, Perth, WA Australia; 18 Department of Intensive Care, Sir Charles Gairdner Hospital, Perth, WA Australia; 19 Emergency Department, Armadale Health Service, Perth, WA Australia; 20 Emergency Department, Sir Charles Gairdner Hospital, Perth, WA Australia; 21 Department of Intensive Care, Gold Coast University Hospital, Gold Coast, QLD Australia

**Keywords:** Sepsis, Fluid therapy, Hypotension

## Abstract

**Background:**

Guidelines recommend an initial intravenous (IV) fluid bolus of 30 ml/kg isotonic crystalloid for patients with sepsis and hypotension. However, there is a lack of evidence from clinical trials to support this. Accumulating observational data suggest harm associated with the injudicious use of fluids in sepsis. There is currently equipoise regarding liberal or restricted fluid-volume resuscitation as first-line treatment for sepsis-related hypotension. A randomised trial comparing these two approaches is, therefore, justified.

**Methods/design:**

The REstricted Fluid REsuscitation in Sepsis-associated Hypotension trial (REFRESH) is a multicentre, open-label, randomised, phase II clinical feasibility trial. Participants will be patients presenting to the emergency departments of Australian metropolitan hospitals with suspected sepsis and a systolic blood pressure of < 100 mmHg, persisting after a 1000-ml fluid bolus with isotonic crystalloid. Participants will be randomised to either a second 1000-ml fluid bolus (standard care) or maintenance rate fluid only, with the early commencement of a vasopressor infusion to maintain a mean arterial pressure of > 65 mmHg, if required (restricted fluid). All will receive further protocolised fluid boluses (500 ml or 250 ml, respectively), if required during the 6-h study period. The primary outcome measure is total volume administered in the first 6 h. Secondary outcomes include fluid volume at 24 h, organ support ‘free days’ to day 28, 90-day mortality, and a range of feasibility and process-of-care measures. Participants will also undergo serial measurement, over the first 24 h, of biomarkers of inflammation, endothelial cell activation and glycocalyx degradation for comparison between the groups.

**Discussion:**

This is the first randomised trial examining fluid volume for initial resuscitation in septic shock in an industrialised country. A pragmatic, open-label design will establish the feasibility of undertaking a large, international, multicentre trial with sufficient power to assess clinical outcomes. The embedded biomarker study aims to provide mechanistic plausibility for a larger trial by defining the effects of fluid volume on markers of systemic inflammation and the vascular endothelium.

**Trial registration:**

Australia and New Zealand Clinical Trials Registry, ID: ACTRN12616000006448. Registered on 12 January 2016.

**Electronic supplementary material:**

The online version of this article (doi:10.1186/s13063-017-2137-7) contains supplementary material, which is available to authorized users.

## Background

Sepsis is defined as life-threatening organ dysfunction caused by a dysregulated host response to infection [1]. A proportion of patients with sepsis develop hypotension, due to such factors as vasodilation and myocardial depression, that can lead to impaired tissue perfusion [2]. The term ‘septic shock’ has been used to describe a state of ‘acute circulatory failure’ in the setting of infection [3]. In the recently updated Third International Consensus Definitions for Sepsis and Septic Shock (Sepsis-3), this term is reserved for the subset of patients in which the underlying circulatory and cellular metabolism abnormalities are such as to substantially increase mortality [1]. Recognising the importance of cellular and microcirculatory alterations in sepsis pathogenesis, a threshold blood pressure is no longer defined for septic shock. Notwithstanding these changes in nomenclature, the conventional initial clinical approach to the sepsis patient with evidence of hypotension/hypoperfusion, as recommended by the Surviving Sepsis Campaign (SSC) guidelines, remains rapid intravenous (IV) infusion of 30 ml/kg of isotonic crystalloid [4]. The rationale for this approach is to restore circulating volume, and to increase stroke volume by optimising cardiac preload. There is no clinical trial data to support this, however, and advances in our understanding of fluid physiology in critical illness have challenged the underlying assumptions [5].

### Challenges to conventional approach

Historically, ‘septic shock’ was understood in simple terms to be synonymous with tissue hypoperfusion; however, the syndrome proves to be far more complex than this. Several lines of evidence challenge this over-simplification. For example:

- Cardiac output may be preserved, increased or depressed in patients with sepsis, although there is a paucity of human studies undertaken in the ‘non-resuscitated’ state [6]
- IV fluid loading has little, if any, effect on cardiac output in critical illness [7], and any increase in blood pressure is not sustained [8]
- Cellular hypoxia is not observed in experimental models of sepsis or in clinical studies [9–11]
- Elevated serum lactate in sepsis is produced aerobically, probably in response to adrenergic stimulation, as an adaptive response to increase bio-energetic efficiency, rather than because of tissue hypoxia [12]
- Organ failure in sepsis involves cellular dysfunction unrelated to hypoxia/hypoperfusion, including structural mitochondrial changes and reduced oxygen consumption [13]
- A key role is played by the microcirculation, including the vascular endothelium, in the pathogenesis of sepsis. These microcirculatory alterations are not related to macrocirculatory indices such as blood pressure [14]

It has been suggested that sepsis with hypotension and/or elevated serum lactate is not strictly a ‘shock’ state, but rather an adaptive alteration in haemodynamics and metabolism [5]. For these reasons we have utilised the term ‘sepsis-associated hypotension’ rather than ‘septic shock’ to denote the clinical phenotype of interest in this trial. Regardless of terminology, attempting to increase cardiac output by administering a fixed, large volume of fluid intravenously does not have a sound theoretical basis.

### Is liberal intravenous fluid administration associated with harm?

Accumulating observational evidence links the injudicious use of intravenously administered fluids with adverse outcome in sepsis, including requirement for organ support and mortality [15–17]. The Fluid Expansion As Supportive Therapy (FEAST) trial randomised 3400 children (median age 24 months) with sepsis and evidence of hypoperfusion to receive bolus resuscitation with either crystalloid saline or albumin, and a control arm whose participants received only maintenance fluid without bolus [18]. Despite early improvement in indices of perfusion, the mortality rate at 48 h was 50% greater in both fluid-bolus groups compared to no bolus (10.6% versus 7.3%). Further analysis found that the excess deaths were due to cardiovascular collapse rather than to the respiratory or neurological complications of fluid administration [19]. However, the trial was undertaken in a resource-poor setting in sub-Saharan Africa. Consequently, there was no access to invasive organ support, over half the participants had malaria and one third were severely anaemic. These factors prevent translation of the results to adults in industrialised countries with ready availability of intensive care. Nonetheless, the FEAST trial is currently the only randomised clinical trial of fluid-bolus resuscitation in sepsis, and has prompted a critical re-evaluation of this approach.

### Why might intravenously administered fluid be harmful?

There are a number of plausible mechanisms by which the inappropriate administration of fluids intravenously may cause harm:

- Tissue oedema – leading to increased requirement for ventilatory support, increased translocation of gut organisms, and increased renal venous pressure compromising perfusion [5]
- Opening of shut-down capillary beds (so called ‘hibernating circulation’) leading to ‘flooding’ of the systemic circulation with cytokine-rich blood, exacerbating systemic inflammation [5]
- Degradation of the glycocalyx layer lining the luminal wall of the vascular endothelium [20]. The glycocalyx plays a critical role in vascular homeostasis by maintaining endothelial cells in a quiescent state. Loss of integrity of the glycocalyx is a critical step in endothelial cell activation and propagation of the systemic inflammatory state. Glycocalyceal damage occurs as a direct effect of fluid administration intravenously [21]

### Alternative approach

Vasopressor medications, such as noradrenaline, have been used for decades to increase vascular tone and reverse the vasodilation and reduced afterload that characterises the ‘classic’ distributive picture of septic shock. Typically, a vasopressor infusion is utilised as a second-line approach to reverse critical hypotension that remains despite IV fluid loading of at least 30 ml/kg, as per SSC guidelines [4]. The optimal timing of vasopressor commencement remains a matter of clinical judgement; like fluids, this specific question has not been the subject of a randomised trial in sepsis. Concerns about the potential harmful effects of vasopressor use in patients who are ‘inadequately fluid-resuscitated’, coupled with the operational requirements that such drugs are administered via a central venous line, may lead to reluctance to utilise vasopressors in a timely fashion. Conversely, many patients with sepsis-associated hypotension ‘respond’ to fluid administration intravenously, at least initially, and the requirement to start vasopressors and admit to a scarce critical care bed may be avoided. These concerns are tempered by emerging evidence of the safety of short-term peripheral administration of vasopressors [22]. In addition, given in low doses, noradrenaline exerts effects on venous capacitance thereby increasing cardiac preload and acting as a physiological ‘volume challenge’ without the need for the administration of exogenous fluids [23].

### Rationale for a randomised trial and the need for pilot data

Excessive fluid administration leads to worse outcomes, and the only clinical trial data demonstrates increased mortality with fluid-bolus therapy [18]. However, for generations physicians have been conditioned to prescribe an IV fluid bolus as first-line treatment for shock. Substantial reductions in sepsis mortality seen in the past two decades have been associated with the introduction of protocolised resuscitation for sepsis, including liberal fluid administration. Waechter et al. examined the effect of the volume of intravenously administered fluid, and the timing of initiation of vasopressors on mortality in a retrospective analysis of over 2000 intensive care unit (ICU) patients [24]. They found that early, high-volume resuscitation in the first 6 h, moderate fluid administration over the subsequent 6–24 h and the timely initiation of vasopressors were associated with the lowest mortality. However, it is unknown whether reductions in mortality occurred as a result of, or in spite of, intravenously administered fluids. Thus, at the present time, there is equipoise regarding liberal or conservative fluid-volume resuscitation in patients with sepsis-associated hypotension [25, 26]. Given the frequency of sepsis among patients admitted to ICU, and the ubiquitous use of intravenously administered fluids in these patients, there is a need for high-quality clinical trial data to guide clinicians and optimise patient outcomes. The REFRESH trial will, therefore, compare a protocolised, restricted-volume resuscitation approach (including the early use of vasopressors, if required) with the standard-volume approach recommended by the SSC.

While a large, multicentre clinical trial is required to investigate the impact of this approach on clinically orientated outcomes such as mortality, pilot data is essential to demonstrate the clinical acceptability of the approach, adherence to the protocol, and whether clinically important differences in mean fluid volume can actually be achieved [27]. The REFRESH trial also aims to provide important mechanistic plausibility data via an embedded biomarker study. Collectively, the results will inform the design of a large trial with sufficient power to detect differences in clinical outcomes.

## Methods/design

### Aim, design and setting

This is an investigator-initiated, multicentre, prospective, randomised open-label pilot (phase II) clinical trial. It will be undertaken in the emergency departments (EDs) of a number of Australian hospitals. These include tertiary-referral and urban general hospitals; at the time of submission six sites are open to recruitment with a further two planned. A pragmatic trial design will test the clinical feasibility of recruitment and administration of the trial intervention, along with an embedded laboratory study that will explore mechanistic plausibility. The protocol adheres to the Standard Protocol Items: Recommendations for Interventional Trials (SPIRIT) Statement (Table 1) (see Additional file 1).

**Table 1:** REstricted Fluid REsuscitation in Sepsis-associated Hypotension (REFRESH) trial Standard Protocol Items: Recommendations for Interventional Trials (SPIRIT) protocol summary

Scientific title	Fluid-restricted versus fluid-liberal resuscitation in sepsis; a randomised controlled pilot trial
Short title	REstricted Fluid REsucitation in Sepsis-associated Hypotension (REFRESH) trial
Health condition	Sepsis
Ethics	HREC/15/Austin/486 (Austin Health, Victoria) & HREC/15/114 (South Metropolitan Health Service, WA)
Protocol version	Version 3, October 2016
Funding	*Grants*:
	Emergency Medicine Foundation (EMSS-229R24-2015-KEIJZERS)
	University of WA/University of Queensland Bilateral Research Collaboration Award
	Royal Perth Hospital Medical Research Foundation
	*Industry*:
	Nil
Primary sponsor	Investigator-initiated and -driven study. Centre for Clinical Research in Emergency Medicine, University of Western Australia. Chief investigator and study contact: Dr. Stephen Macdonald, Royal Perth Hospital, PO Box X2213, Perth, WA, Australia
	Email: stephen.macdonald@uwa.edu.au
Background	Sepsis is a common condition with high morbidity and mortality. Patients with sepsis can develop hypotension (low blood pressure) due to a combination of factors. The traditional first-line treatment of sepsis-associated hypotension is to give a rapid, large volume of intravenously administered fluid (fluid bolus). Emerging clinical data and advances in the understanding of fluid physiology suggest that fluid bolus may be associated with worse patient outcomes. This randomised pilot clinical trial will compare a restricted fluid-volume approach, including early use of vasopressor drugs if required, against standard (liberal fluid-volume) care to assess the feasibility and safety of this approach. An embedded laboratory study will measure the differences in a range of relevant blood markers (such as activation of the vascular endothelium) to provide mechanistic plausibility for a restricted-volume approach. Together these data will be used to inform the design of a large multicentre trial to assess clinical outcomes
Hypothesis	That a volume-restricted approach to sepsis-associated hypotension is clinically feasible; that this approach results in reduced systemic inflammation and associated biomarkers of endothelial cell activation
Study aims	1. To investigate the feasibility of delivering volume-restricted resuscitation in sepsis-associated hypotension
	2. Compare the total volume of fluid administered at 6 h and 24 h
	3. To assess effects on the biomarkers of the inflammatory response, endothelial activation and related pathways of interest
	4. To record any differences in requirement for organ support and in clinical outcomes
Study design	• Multi-centre (Armadale, Perth/Austin, Melbourne/Fiona Stanley, Perth/Gold Coast/Royal Hobart/Royal Perth/Sir Charles Gairdner, Perth/The Prince Charles, Brisbane)
	• Randomised controlled/un-blinded/feasibility
	• Interventional
Setting	8 emergency departments (EDs) in Australia
Inclusion and exclusion criteria	*Inclusion criteria*:
	1. Suspected infection *and*
	2. Systolic blood pressure (SBP) < 100 mmHg, despite 1000 ml intravenously administered isotonic crystalloid administered over not more than 60 minutes *and*
	3. Study intervention can be administered within 2 hs of inclusion criteria being met
	*Exclusion criteria*:
	1. Hypotension thought due to, or contributed to by, a non-sepsis cause (e.g. arrhythmia, haemorrhage)
	2. Clinical requirement for fluid replacement (e.g. gastrointestinal losses)
	3. Transfer from another hospital
	4. More than 2000 ml intravenously administered fluid has been given (pre-hospital, in ED, or both)
	5. Likely requirement for immediate surgery
	6. Age < 18 years
	7. Pregnancy (confirmed or suspected)
	8. Patient in extremis or death deemed imminent and inevitable
	9. Patient wishes or comorbidities such that either fluid loading or vasopressor support is not considered clinically appropriate
Intervention	*Intervention arm*:
	Commence vasopressor infusion (± maintenance IV fluid) if SBP < 90 mmHg or mean arterial pressure (MAP) < 65 mmHg). Reassess hourly next 6 h and administer further 250-ml IV fluid bolus if required
	*Comparator arm*:
	Give a second 1000-ml fluid bolus plus further 500-ml boluses as clinically indicated until judged to be euvolaemic. Commence vasopressor infusion (± maintenance IV fluid) if SBP < 90 mmHg or MAP < 65 mmHg). Reassess hourly next 6 h and administer further 500-ml IV fluid bolus if required
Primary outcome measure	Total volume of intravenously administered fluid (including pre-randomisation) at 6 h
Secondary outcome measures	• Mortality (all-cause) at 90 days post enrolment
	• ICU length of stay
	• Hospital length of stay
	• Hospital-free days to day 90
	• Organ failure
	o Cardiovascular
	▪ Requirement for vasopressors
	▪ Duration of vasopressor requirement (h)
	▪ Vasopressor-free days to day 28
	o Respiratory
	▪ Requirement for ventilation (NIV/IPPV)
	▪ Duration of ventilator support (h)
	▪ Ventilator-free days to day 28
	o Renal
	▪ Requirement for renal replacement therapy (RRT)
	▪ Duration of RRT
	▪ RRT-free days to day 28
	▪ AKIN score to day 7
Laboratory/mechanistic outcome measures	Peak values, as well as patterns of expression over time (T0–T24), will be analysed for a range of biomarkers and compared between the groups including:
	• Atrial natriuretic peptide
	• Troponin
	• Inflammatory cytokines
	o IL-6, IL-10, resistin
	• NGAL (a biomarker of renal injury)
	• Endothelial cell activation biomarkers
	o sVCAM, sICAM, sE-Selectin, sFlt-1
	• Soluble markers of glycocalyx degradation
	o Heparan sulphate, syndecan-1, hyaluronan
Feasibility outcome measures	• Proportion of eligible patients enrolled
	• Randomisation errors
	• Compliance – protocol violations
	• Proportion with completely recorded data
	• Proportion with complete study blood sampling
Sample size	50 per arm/100 total
Randomisation	Eligible participants in whom a consent process has been commenced will be randomised to either the fluid-volume restricted or the fluid-liberal (standard-care arm)
Randomisation procedure	• Eligibility assessment and randomisation conducted by clinical staff with support by research personnel (in person or by telephone)
	• Computer-generated randomisation sequence
	• Real-time web-based randomisation
	• 1:1 randomisation in blocks of 2 or 4
Blinding	• Un-blinded to patients and to treating team
	• Blinding of laboratory staff and data analysts
Data collection methods	• Paper CRF completed for each participant
	• CRF populated by research staff in real time or from clinical record
	• Subsequent data entry into secure database by trained research assistant
	• Audit of data entry against source documentation
Data collected	• All fluids administered pre-randomisation
	• All fluids administered post randomisation – 6 h (recorded hourly)
	• All fluids administered over 6–24 h
	• Urine output to 24 h
	• Vital signs at enrolment and hourly until 6 h
	• Antimicrobials – agent, dose and time administered
	• Corticosteroid administration
	• Source of sepsis (suspected/proven)
	• Charlson Comorbidity Score
	• Microbiological/serological results from samples obtained in first 24 h
	• Sequential Organ Failure Assessment (SOFA) score at admission and at 24 h
	• Acute Physiology and Chronic Health Evaluation (APACHE) II score (peak first 24 h)
	• Acute Kidney INjury (AKIN) score up to 7 days
	• Study blood sampling at enrolment (T0), 3 h (T3), 6 h (T6) and 12–24 h (T24)
Statistical analysis	• Intention-to-treat analysis
	• Statistician blinded to treatment allocation
Data and safety monitoring	• DSMC – emergency physician, intensive care physician, statistician
	• SAE reporting to study coordination centre within 24 h
	• DSMC review all SAE
	• Periodic data review by DSMC and recommendations to PI in event of study conduct of SAE issues
	• Trial coordinator will monitor trial conduct at each site at regular intervals
Ethics and governance	• HREC approvals obtained for all sites
	• Clinical governance (site-specific authority) procedures in place for all sites
	• Any subsequent protocol amendments to be submitted to lead ethics site with appropriate dissemination to participating study sites
	• The study will be conducted in accordance with GCP
Consent	• Where possible, prospective informed consent will be obtained
	• Due to the time-critical nature of the condition, approval in place for verbal consent to randomise and initiate care followed by formal written consent process
	• Some participants will lack capacity so consent will be sought from next of kin
	• Where next of kin not immediately available, provision in place for enrolment under initial waiver of consent (or procedural authorisation) followed by delayed consent to continue in the trial
Confidentiality	• All data will be de-identified
	• Data (paper and electronic) will be stored securely
Dissemination	• Trial results to be presented at relevant scientific meetings and published in peer-reviewed journal
Study status	• Opened to recruitment July 2016 (Protocol V2)
	• Protocol amended October 2016 (Protocol V3) after 6 participants enrolled
	• As at 22 June 2017, 42 participants enrolled at 6 of 7 sites active
	• Anticipated to complete enrolment of 100 participants within 12 months

*AKIN* Acute Kidney Injury Network*, CRF* Case Report Form, *DSMC* Data Safety Monitoring Committee*, GCP* Good Clinical Practice, *ICAM* intercellular adhesion molecule, *IL* interleukin, *IPPV* intermittent positive-pressure ventilation; *IV* intravenous, *NGA* neutrophil gelatinase-associated lipocalin, *NIV* non-invasive ventilation, *PI* principal investigator, *SAE* serious adverse event, *VCAM* vascular cell adhesion molecule

### Participant characteristics

The trial will enrol 100 adult patients (aged 18 years or older) with sepsis, according to the recently revised standard consensus definition (Sepsis3) [1], presenting to the ED of a participating hospital, and who have a systolic blood pressure (SBP) < 100 mmHg, persisting despite intravenous administration of a fluid bolus.

#### Inclusion criteria

1. Suspected infection *and*
2. Systolic blood pressure (SBP) < 100 mmHg*, despite 1000 ml isotonic crystalloid administered intravenously over not more than 60 mins *and*
3. Study intervention can be administered within 2 h of inclusion criteria being met

#### Exclusion criteria

1. Hypotension thought due to, or contributed to by, a non-sepsis cause (e.g. arrhythmia, haemorrhage)
2. Clinical requirement for fluid replacement (e.g. gastrointestinal losses)
3. Transfer from another hospital
4. More than 2000 ml* of intravenously administered fluid has been given (pre-hospital, in ED, or both)
5. Likely requirement for immediate surgery
6. Age below 18 years
7. Pregnancy (confirmed or suspected)
8. Patient in extremis or death deemed imminent and inevitable
9. Patient wishes or comorbidities such that either fluid loading or vasopressor support is not considered clinically appropriate

*The previous version of the protocol (V2, dated April 2016) required a systolic blood pressure < 90 mmHg for inclusion, and excluded patients who had received > 1000 ml of fluid. These criteria were amended because of a slow recruitment rate. A total of six participants were enrolled under this protocol, all of which meet eligibility for the trial in the current version.

### Screening and randomisation

Patients with clinical features of infection will be screened for sepsis using standard clinical procedures including recording of vital signs, and collection of blood samples including full blood count, urea and electrolytes and a venous blood gas analysis including serum lactate. Those who have a systolic blood pressure < 100 mmHg on arrival, or who develop this during their ED stay, will receive a 1000-ml IV fluid challenge with isotonic crystalloid over a maximum of 60 min. Patients whose blood pressure remains < 100 mmHg, or whose blood pressure falls below this threshold within 60 min of completing the fluid challenge, and who do not have any exclusion criteria will be invited to participate in the trial. Participants who fulfil the eligibility criteria and for whom consent is obtained (see ‘Ethics and consent’ below) will be randomised (1:1) to one of the study arms. The treating clinician will perform randomisation in real time using a dedicated, secure, password-protected web-based interface. Allocation concealment will be maintained until the conclusion of the online randomisation process. Randomisation, using a computer-generated random sequence, will be stratified by site in pre-allocated blocks of 2 and 4, thus making it impossible to predict the last allocation in a block. For practical and safety purposes randomised trial participants and the treating team will not be blind to the trial intervention. The staff conducting the laboratory analyses and the trial statistician will be blinded to the treatment allocation. The screening and enrolment procedure is summarised in (see Additional file 2: Figure S1).

### Study interventions

#### General management – all participants

1. Supplemental oxygen to maintain SpO_2_ > 92%
2. Antibiotics within 60 min of enrolment
3. Ventilation (non-invasive) or intubation and mechanical ventilation, if clinically indicated, using a lung preventive strategy as recommended by SSC guidelines [4]; consider ketamine for induction to minimise risk of hypotension
4. All fluids administered (bolus or maintenance) subsequent to enrolment to be balanced isotonic crystalloid during the first 6 h. Hypotonic fluids and synthetic colloids and 0.9% saline are to be avoided. Blood products and albumin may be given at treating clinician discretion. Actual (unadjusted) volumes of all non-crystalloid fluids will be recorded
5. Fluid management will be as per study protocol for the first 6 h post randomisation regardless of whether the patient remains in the ED or is transferred to ICU or to a ward. For patients with an unanticipated transfer to theatre for surgery during this time the trial protocol will be suspended

#### Restricted-volume (±early vasopressor) arm (see Additional file ***3***: Figure S2)

Management following randomisation after the initial 1000-ml fluid bolus for the next 6 h:

1. Continue fluid infusion intravenously at a *maintenance rate* of 1–2 ml/kg/h (max 150 ml/h) via infusion pump, as clinically indicated
2. If SBP < 90 mmHg or mean arterial pressure (MAP) < 65 mmHg:
  - Start noradrenaline* infusion by peripheral IV route (large vein) and titrate to maintain MAP at 65–70 mmHg
  - Insertion of urinary catheter and hourly urine output measures
  - Insertion of central venous access (central venous catheter (CVC)/ peripheral intravenous central catheter (PICC)) and commencement of central noradrenaline infusion once correct position confirmed by chest X-ray. Alternatively, at physician discretion and according to local protocol, if the anticipated duration of vasopressor requirement is less than 6 h the infusion can continue via a dedicated secure peripheral large venous cannula if the patient is monitored in a critical care environment and there is no other indication for central access
  - Arterial line at the discretion of the treating clinician
  - Titration and weaning of vasopressors as per treating clinician and local protocols
  - Additional therapies, e.g. vasopressin, corticosteroids at the treating clinician’s discretion
  - If, in the judgement of the treating clinician (based upon clinical assessment, central venous pressure (CVP), ultrasound, etc.), the patient remains hypovolaemic, a further 1000-ml isotonic crystalloid bolus is permitted
3. Reassess each hour for the first 6 h. At each reassessment consider need for further fluid bolus based upon clinical assessment, including:
  - Mental status, skin perfusion
  - Urine output
  - Repeat lactate (if initially elevated)
  - Ultrasound/echocardiography or CVP if available

If the treating clinician judges additional fluid to be required, administer an additional *250-ml* isotonic crystalloid IV bolus.

#### Standard treatment arm (see Additional file ***4***: Figure S3)

Management following randomisation after the initial 1000-ml fluid bolus for the next 6 h:

1. Give a *second fluid bolus* of 1000 ml (balanced isotonic crystalloid)
2. Reassess
  - If SBP ≥ 90 mmHg and MAP ≥ 65 mmHg give maintenance rate of 1–2 ml/kg/h (max 150 ml/h) via infusion pump, as clinically indicated
  - If SBP < 90 mmHg or MAP < 65 mmHg:
    i. Give further *500-ml* boluses, at least every 30 min, until the treating clinician judges euvolaemia to be achieved
    ii. Once euvolaemia is deemed present (e.g. inferior vena cava (IVC) non-collapsing on ultrasound, CVP > 8 mmHg, distended neck veins, etc.) and if the patient remains hypotensive:
      1. Start noradrenaline* infusion by peripheral IV route (large vein) and titrate to maintain MAP at 65–70 mmHg
      2. Insertion of urinary catheter and hourly urine output measures
      3. Insertion of a central venous access (CVC/PICC) and commencement of central noradrenaline infusion once correct position confirmed by chest X-ray. Alternatively, at physician discretion and according to local protocol, if the anticipated duration of vasopressor requirement is less than 6 h the infusion can continue via a dedicated secure peripheral large venous cannula if the patient is monitored in a critical care environment and there is no other indication for central access
      4. Arterial line at the discretion of the treating clinician
      5. Titration and weaning of vasopressors as per treating clinician and local protocols
      6. Additional therapies, e.g. vasopressin, corticosteroids at the treating clinician’s discretion
3. Reassess each hour for the first 6 h. At each reassessment consider the need for further fluid boluses based upon clinical assessment, including:
  - Mental status, skin perfusion
  - Urine output
  - Repeat lactate (if initially elevated)
  - Ultrasound/echocardiography or CVP if available

If the treating clinician judges additional fluid to be required, administer an additional *500-ml* isotonic crystalloid IV bolus.

*An infusion of metaraminol via a peripheral IV cannula may be considered as an alternative to noradrenaline in accordance with local hospital policy.

#### Study measurements

##### Clinical

- All fluids administered pre-randomisation
- All fluids administered post-randomisation – 6 h (recorded hourly)
- All fluids administered over 6–24 h
- Urine output to 24 h
- Vital signs at enrolment and hourly until 6 h
- Antimicrobials – agent, dose and time administered
- Corticosteroid administration
- Source of sepsis (suspected/proven)
- Charlson Comorbidity Score [28]
- Microbiological/serological results from samples obtained in first 24 h
- Sequential Organ Failure Assessment (SOFA) score [29] at admission and at 24 h
- Acute Physiology and Chronic Health Evaluation (APACHE) II score [30] (peak first 24 h)

##### Laboratory

Study blood sampling:

- Enrolment (T0), 3 h (T3), 6 h (T6) and 12–24 h (T24)
- Plasma and serum samples (total 5 ml) at each sampling time point
- Blood gas (arterial or venous) including lactate and haemoglobin at each sampling time point
- Blood samples should not to be taken during fluid-bolus administration and should be taken from the opposite arm from any maintenance fluid being administered

Samples will be centrifuged and the extracted sera/plasma divided into 500-μl aliquots and stored at −80 °C for subsequent batch analysis.

### Strategies to ensure protocol compliance and data integrity

Digital copies of de-identified Case Report Forms will be submitted electronically to the trial coordination centre at the following time points; enrolment, completion of hospital stay, 28 days and 90 days. This will allow for checking and data queries to be undertaken in a timely manner. This will also identify any protocol deviations and provide opportunity for feedback to the site investigator. A trained research assistant at the trial coordination centre will upload data into a secure trial database. There will be periodic internal audits of data accuracy and compliance.

### Outcome measures

Consistent with the exploratory pilot nature of this study, multiple feasibility, process-of-care, clinical and mechanistic outcomes will be evaluated. The primary outcome relates to achieving a difference in the volume of intravenously administered fluid to the groups. All analyses will be by intention-to-treat.

#### Primary outcome

- A clinically and statistically significant difference in mean total fluid volume administered at 6 h post randomisation (including pre-randomisation). In addition pre- and post-randomisation volumes will be reported separately. See ‘Statistical methods and sample size calculation’ below.

#### Secondary outcomes: feasibility

- Proportion of screened patients eligible
- Proportion of eligible patients enrolled
- Enrolment rate (i.e. number of enrolments per month per site)
- Protocol compliance

#### Secondary outcomes: process-of-care

- Admission rate to ICU versus general ward
- Rates and timing of CVC insertion
- Peripheral versus central administration of vasopressors

#### Secondary outcomes: clinical

- Mortality (all-cause) at 90 days post enrolment
- ICU length of stay
- Hospital length of stay (acute)
- Hospital-free days to day 90 (acute)
- Organ failure

- o Cardiovascular
  - ▪ Administration of vasopressors
  - ▪ Duration of vasopressor requirement (h)
  - ▪ Vasopressor-free days to day 28
- o Respiratory
  - ▪ Requirement for ventilation (non-invasive ventilation (NIV)/intermittent positive-pressure ventilation (IPPV))
  - ▪ Duration of ventilator support (h)
  - ▪ Ventilator-free days to day 28
- o Renal
  - ▪ Requirement for renal replacement therapy (RRT)
  - ▪ Duration of RRT
  - ▪ RRT-free days to day 28
  - ▪ Acute Kidney Injury Network (AKIN) score to day 7 [31]

#### Laboratory/mechanistic outcomes

Peak values, as well as patterns of expression over time (T0–T24), will be analysed for a range of biomarkers and compared between the groups including:

- Atrial natriuretic peptide
- Troponin
- Inflammatory cytokines
  - o Interleukin (IL)-6, IL-10, resistin
- Neutrophil gelatinase-associated lipocalin (NGAL; a biomarker of renal injury)
- Endothelial cell activation biomarkers
  - o sVCAM, sICAM, sE-Selectin, sFlt-1
- Soluble markers of glycocalyx degradation
  - o Heparan sulphate, syndecan-1, hyaluronan

All laboratory analyses will be performed and the results collated by laboratory staff blinded to the study group allocation. As a pragmatic, open-label trial, treating clinicians and participants will be aware of the treatment allocation.

**Fig. 1:**
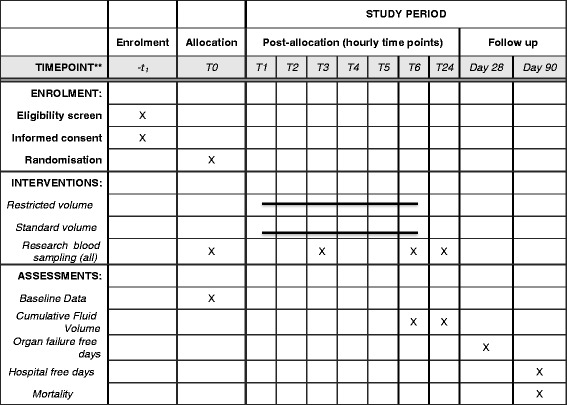
Standard Protocol Items: Recommendations for Interventional Trials (SPIRIT) Figure

The schedule of enrolment, interventions and assessments is shown in Fig. 1.

### Statistical methods and sample size calculation

The primary outcome is the achievement of a minimum clinically significant relative difference of 33% in the total cumulative fluid volume administered until T6 (including pre-randomisation fluids). In the control arm of an Australasian, multicentre randomised controlled trial of early goal-directed therapy in septic shock, the mean total volume administered within the first 6 h was 4200 ± 2650 ml [32]. We aim to achieve a reduction of 33%, to a mean of 2800 ± 1800 ml over 6 h in the restricted-volume arm compared to the standard-care arm. This will require 43 participants in each group to achieve 80% power with *α* = 0.05. This pilot trial is not designed to have sufficient power to detect differences in secondary clinical outcomes.

The trial will also measure a range of biochemical markers. There is limited relevant data to inform sample size calculations for these analyses. Based upon feasible recruitment within a 12-month time period, a sample size of 100 is attainable. This will allow a margin of safety for achieving differences in the primary outcome. It will also enable the detection of differences between means of the two treatment groups of 55.6% of the standard deviation (or larger) for the measured biomarker, with 80% power and *α* = 0.05. This calculation is based upon a cross-sectional *t* test, not a repeated measures test (which affords additional power). As such, these calculations are conservative.

Baseline clinical and demographic data will be analysed using chi-square or Fisher’s exact test for categorical data, and the Wilcoxon rank-sum test for continuous data.

Where biomarkers are normally or log-normally distributed, linear mixed models with maximum likelihood estimation will be employed to analyse the pattern over time. Differences in the pattern of expression of each biomarker between the groups will be tested by the interaction of time and group in the model. Additional covariates will be added to adjust for treating centre, illness severity and comorbidities. For biomarkers with skewed distributions resistant to successful transformation, quantile regression with per-person cluster adjustment will be used.

A biostatistician will perform the statistical analysis.

### Trial administration and Data Safety Monitoring Committee

Trial coordination will be by the Centre for Clinical Research in Emergency Medicine (CCREM) at the University of Western Australia, Perth, Australia. Case Report Forms will be completed by site investigators and sent electronically (after removal of individual patient identifiers) to the trial coordination centre for checking and data entry into a purpose-designed secure Research Electronic Data Capture (REDCap – Vanderbilt University, Nashville, TN, USA) database hosted by the University of Western Australia. Study blood samples will be processed at local hospital laboratories and stored for subsequent bulk shipping to the coordinating centre. All biomarker analyses will be performed in house at the CCREM laboratory facility in Perth.

The trial will be overseen by a Steering Committee, comprising site investigators from each participating site. The Steering Committee will meet monthly by teleconference. An independent Data Safety Monitoring Committee (DSMC) comprising an emergency physician, intensive care physician and biostatistician has been appointed to oversee the trial and will undertake periodic reviews at the discretion of the chair. The DSMC will review all deaths and adverse events (see below) and have the authority to suspend or halt recruitment if necessary. The DMSC will also assess relevant emerging published academic literature favouring one of the arms of the trial. Due to the small numbers precluding determination of any statistically significant difference in clinical outcomes between the arms, no formal interim analysis will be undertaken and no stopping rules are specified.

### Adverse events

Adverse events (AEs) are defined as any untoward medical occurrence in a patient administered an investigational intervention which does not necessarily require to have a causal relationship with this intervention.

Patients with severe sepsis in the ED and ICU commonly have aberrations in laboratory values, signs and symptoms due to the severity of their underlying disease and the impact of standard therapies [33]. These will not necessarily constitute an AE unless they require significant amelioration or are considered to be of concern in the investigator’s clinical judgment. AEs include all unexpected and/or untoward medical events experienced by the patient *which are not part of the expected course of sepsis*.

The following will be considered AEs, regardless of study treatment allocation and subsequent intervention required:

- Extravasation of peripherally administered noradrenaline infusion (whether or not any tissue injury occurred)
- CVC-related complications
  - Bleeding
  - Inadvertent arterial puncture
  - Pneumothorax
  - Thrombosis
  - Infection
  - Arterial catheter-related complications, including thrombosis
  - Any other event that is considered to be of concern by the site investigator

The AEs listed above will be recorded from the time of commencement of study treatment to 72 h post intervention. CVC-related complications, including infection, will be considered an AE for as long as the CVC remains in situ.

All AEs will be reported, irrespective of treatment allocation to the chief investigator and/or the REFRESH Management Committee and then forwarded to the chair of the DSMC.

### Serious adverse events

A serious adverse event (SAE) is defined as any untoward medical occurrence that:

- Results in death
- Is life-threatening
- Requires inpatient hospitalisation or prolongation of existing hospitalisation
- Results in persistent or significant disability/incapacity
- Is an important medical event, which may require intervention to prevent one of the previously listed outcomes

In this study, all SAEs will be reported regardless of suspected causality.

### Adverse event and serious adverse event reporting

Separate Case Report Forms will be developed to record AEs and SAEs. SAEs occurring from the time of commencement of study treatment to 72 h post intervention will be reported to the coordinating centre by faxing the supplied SAE Form within 24 h of site study staff becoming aware of the event. In this pilot trial, all deaths up to day 30 will be reported as a SAE. Coordinating centre staff will be responsible for following up SAEs to ensure that all details are available. It is the responsibility of each site to inform their Human Research Ethics Committee (HREC) of all SAEs which occur at their site, in accordance with local requirements.

All SAEs will be forwarded to the chair of the DSMC within 24 h of receipt at the REFRESH coordinating centre. As detailed in the Patient Information and Consent Form, any injury or complication occurring as a result of trial participation is to be reported to the study team who will arrange all necessary medical treatment. In Australia, Medicare will provide any required medical treatment, free of charge, in a public hospital.

### Strategies to achieve adequate enrolment

An education package has been developed for staff at enrolling sites. In addition, individual site visits (physical or virtual) will be undertaken at trial commencement. The Trial Steering Committee will meet by teleconference at least monthly and review enrolment rates and discuss strategies to maximise these. Several sites have dedicated research nurses in the ED who will actively screen for eligibility. Finally, the trial will be publicised by presentations at academic meetings and via social media platforms – e.g. Twitter @REFRESH_Trial.

## Discussion

### Significance

This will be the first randomised trial examining the question of fluid volume in the early resuscitation of adult patients with sepsis-associated hypotension in a mature health care system with access to advanced critical care. This is an important clinical question given the findings of observational studies and the FEAST trial [18]. Given the universal use of fluids to manage this condition, superiority of a restricted approach is likely to have significant clinical and economic impact. A recent trial demonstrated the feasibility of a restricted-volume approach to sepsis in ICU [34]. However, the median volume of fluid administered prior to randomisation was over 4 l. By recruiting patients in the ED, at the earliest point in the course of their care, we aim to investigate very early resuscitation. This essentially replicates the approach of the FEAST trial [18], but in an industrialised country with access to advanced critical care.

Undertaking such a trial in the ED setting presents considerable challenges. Unlike the ICU, patients present to the ED with undifferentiated illness. Sepsis is a highly heterogeneous condition. Often the onset of illness is insidious, and the clinical presentations highly variable. In addition, sepsis is a clinical syndrome and there is a lack of a standard diagnostic test. Nevertheless, the ARISE trial [32] demonstrated that the identification and enrolment of patients with sepsis into a clinical trial in Australian EDs is possible.

The REFRESH trial will provide essential feasibility and mechanistic plausibility data to support a large-scale clinical trial. A multicentre pilot will allow assessment of the feasibility of delivering the protocol in a range of settings. For example, participating sites are inclusive of both tertiary referral centres and urban general hospitals. A number of sites have substantial research infrastructure, including research nurses within the ED, whereas at other sites the trial will rely on enrolments by clinical staff. Metrics, such as rates of recruitment of eligible patients and adherence to the trial protocol, will be assessed as part of the feasibility objectives. All this information will be utilised in designing the optimal protocol for a trial that is clinically acceptable and operationally achievable.

### Limitations

An open-label design confers challenges but is unavoidable given the nature of the trial intervention. Potential bias arises due to the treating clinician being aware of the treatment allocation. The pragmatic nature of the trial allows for some latitude for the clinician to determine fluid volumes within set parameters; however, it is difficult to control for individual behaviours and treating physician preference for one approach over another. An additional potential challenge may be the reluctance to give fluid in the standard-care arm on the basis of perceived potential harms (the rationale for the trial). To counter this, regular feedback and educational sessions with clinicians will occur throughout the trial. For this pilot trial, the primary feasibility outcomes are objective measures (e.g. fluid volumes, processes of care). Clinical outcome measures are also objective and pre-specified, e.g. mortality, organ failure. However to minimise any potential risk of bias, a physician-investigator will adjudicate clinical outcomes blinded to group allocation. Similarly, laboratory analyses will be conducted in blinded fashion.

### Stakeholder engagement and dissemination of findings

The investigator team comprise clinicians in emergency medicine and intensive care who have substantial experience in conducting research among critically ill patients. The REFRESH protocol has been developed over an 18-month period during which extensive consultations took place. The protocol has been informed by practical feedback including from clinician groups locally at participating sites, and following presentation at the Australian and New Zealand Intensive Care Society Clinical Trials Group (CTG), and the Annual Scientific Meeting of the Australasian College for Emergency Medicine (ACEM). The REFRESH trial has received formal endorsement from the ACEM CTG.

The trial results will be of wide interest to the global emergency and critical care communities. It is anticipated that the results will be used to inform the design of, and secure funding for, a large-scale clinical trial to evaluate patient-centred outcomes. The results of the trial will be submitted for publication in a suitable international journal. A writing committee will be convened from among the Trial Steering Committee. Authorship criteria will be according to the International Committee of Medical Journal Editor definition.

### Trial status

At the time of submission the REFRESH trial has enrolled 42 of its planned 100 participants.

## Additional files

Additional file 1: SPIRIT 2013 Checklist: recommended items to address in a clinical trial protocol and related documents*. (DOC 120 kb)

Additional file 2: Figure. Screening and enrolment flowchart. (PDF 422 kb)

Additional file 3: Figure. Restricted fluid volume arm. (PDF 497 kb)

Additional file 4: Figure. Standard fluid volume arm. (PDF 419 kb)

Additional file 5: Appendix. Verbal consent script. (DOCX 131 kb)

Additional file 6: Figure. REFRESH trial consent procedure. (PDF 400 kb)

## Abbreviations

ACEM: Australasian College for Emergency Medicine
AE: Adverse event
AKIN: Acute Kidney Injury Network
APACHE: Acute Physiology And Chronic Health Evaluation
ARISE: Australasian Resuscitation in Sepsis Evaluation
CVC: Central venous catheter
DSMC: Data Safety Monitoring Committee
ED: Emergency department
FEAST: Fluid expansion as supportive therapy
HREC: Human Research Ethics Committee
ICAM: Intercellular adhesion molecule
ICU: Intensive care unit
IL-6/10: Interleukin-6/10
IPPV: Intermittent positive-pressure ventilation
IV: Intravenous
MAP: Mean arterial pressure
NGAL: Neutrophil gelatinase-associated lipocalin
NIV: Non-invasive ventilation
PICC: Peripheral intravenous central catheter
SAE: Serious adverse event
SBP: Systolic blood pressure
SOFA: Sequential Organ Failure Assessment
SpO_2_: Peripheral transcutaneous oxygen saturation
SSC: Surviving Sepsis Campaign
VCAM: Vascular cell adhesion molecule
