# Supplementary figures and images for: REstricted Fluid REsuscitation in Sepsis-associated Hypotension (REFRESH): study protocol for a pilot randomised controlled trial
Source: Trials. 2017 Aug 29;18:399. doi: 10.1186/s13063-017-2137-7 (PMC5576288; doi:10.1186/s13063-017-2137-7)

# REFRESH

## Enrolment flowchart

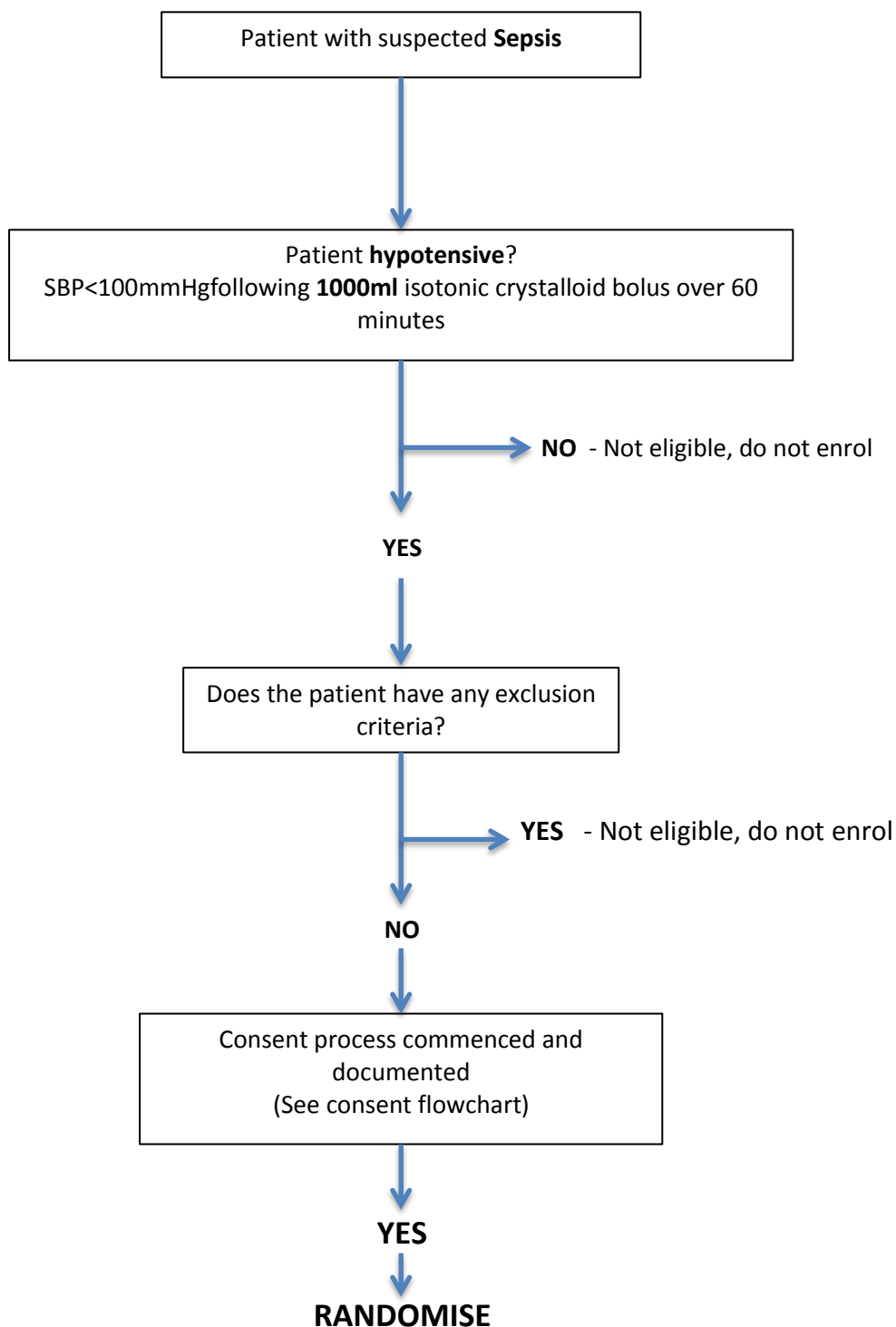

Supplement: Supplementary file 2 — Figure. Screening and enrolment flowchart. (PDF 422 kb) [file 13063_2017_2137_MOESM2_ESM.pdf]

# REFRESH

## Restricted volume/early vasopressor arm

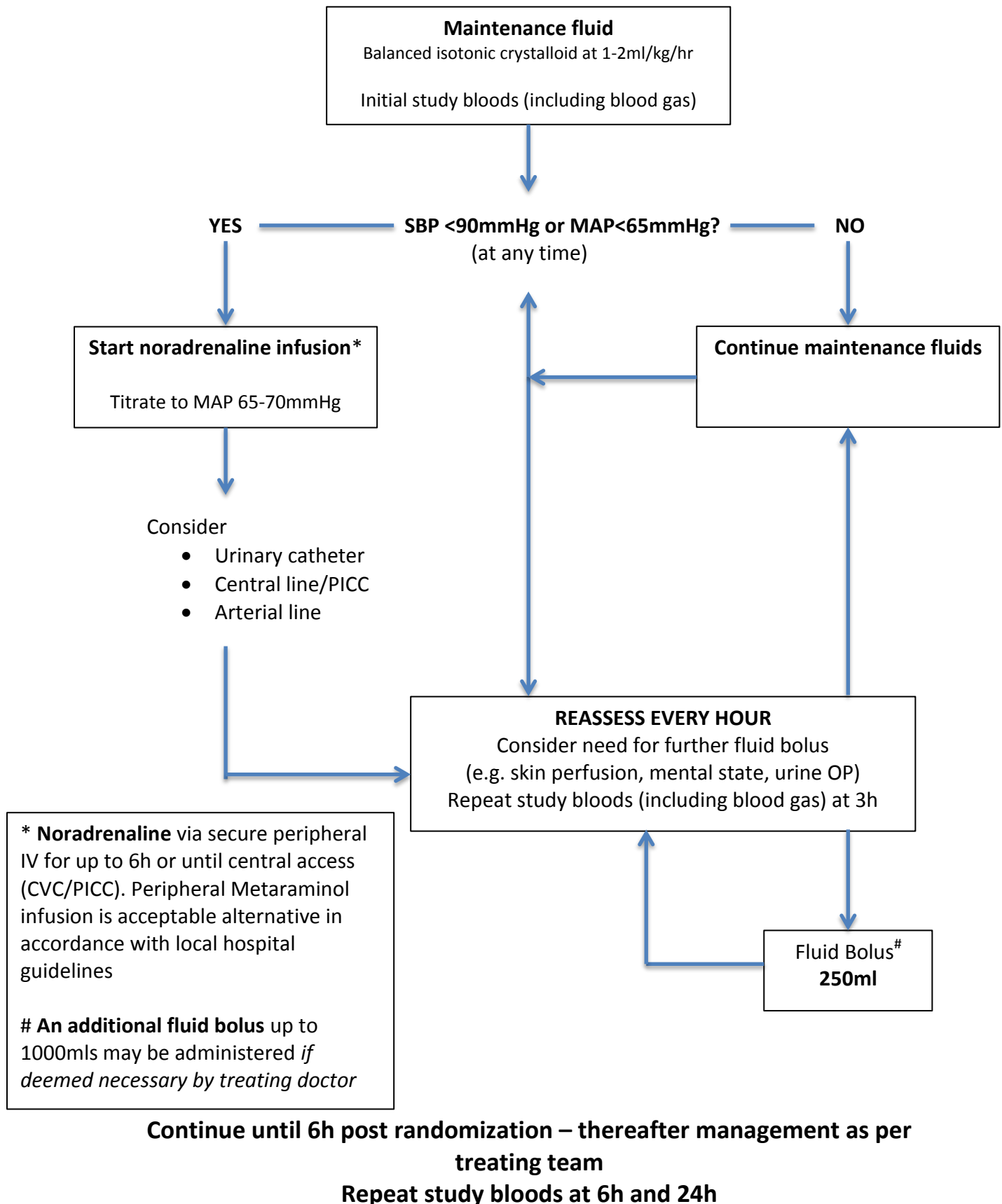

Supplement: Supplementary file 3 — Figure. Restricted fluid volume arm. (PDF 497 kb) [file 13063_2017_2137_MOESM3_ESM.pdf]

# REFRESH

## Consent Process

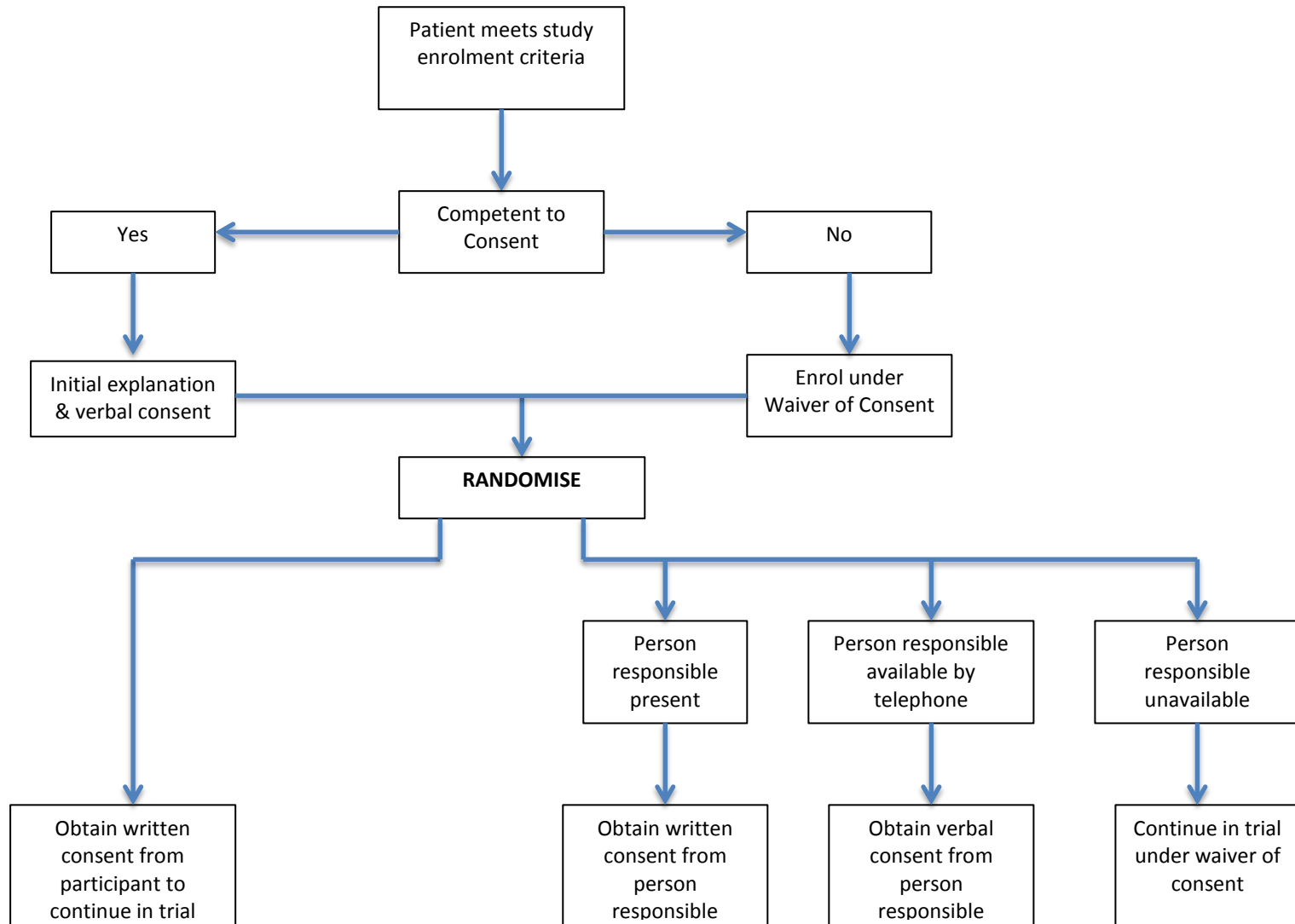

Supplement: Supplementary file 6 — Figure. REFRESH trial consent procedure. (PDF 400 kb) [file 13063_2017_2137_MOESM6_ESM.pdf]
