# Supplementary material for: REstricted Fluid REsuscitation in Sepsis-associated Hypotension (REFRESH): study protocol for a pilot randomised controlled trial
Source: Trials. 2017 Aug 29;18:399. doi: 10.1186/s13063-017-2137-7 (PMC5576288; doi:10.1186/s13063-017-2137-7)
Supplement: Supplementary file 4 — Figure. Standard fluid volume arm. (PDF 419 kb) [file 13063_2017_2137_MOESM4_ESM.pdf]

# REFRESH

## Standard volume arm

**Second fluid bolus 1000ml**
Balanced isotonic crystalloid

Initial study bloods (including blood gas)

**YES** — **SBP <90mmHg or MAP<65mmHg?** — **NO**
(at any time)

**Give further 500mls fluid boluses at least every 30 min until euvolaemic**

If persistent SBP<90mmHg or MAP <65mmHg
**Start noradrenaline infusion***
Titrate to MAP 65-70mmHg

Consider
- Urinary catheter
- Central line
- Arterial line

**Commence maintenance fluids**
Balanced isotonic crystalloid at 1-2ml/kg/hr

**REASSESS EVERY HOUR**
Consider need for further fluid bolus
(e.g. skin perfusion, mental state, urine OP)
**Repeat study bloods (including blood gas) at 3h**

Fluid Bolus
**500ml**

* **Noradrenaline** via secure peripheral IV for up to 6h or until central access (CVC/PICC). Peripheral Metaraminol infusion is acceptable alternative in accordance with local hospital guidelines

**Continue until 6h post randomization – thereafter management as per treating team**
**Repeat study bloods at 6h and 24h**
