# Supplementary material for: REstricted Fluid REsuscitation in Sepsis-associated Hypotension (REFRESH): study protocol for a pilot randomised controlled trial
Source: Trials. 2017 Aug 29;18:399. doi: 10.1186/s13063-017-2137-7 (PMC5576288; doi:10.1186/s13063-017-2137-7)
Supplement: Supplementary file 5 — Appendix. Verbal consent script. (DOCX 131 kb) [file 13063_2017_2137_MOESM5_ESM.docx]

**APPENDIX - REFRESH trial verbal script for initial consent to randomise**

We are doing some research to find out the best way to treat patients with your condition. Your doctor thinks you have an infection and this is causing you to have low blood pressure. The usual treatment for this is to give intravenous fluids. If blood pressure does not improve, medication is sometimes required to achieve this.

There is currently debate about the right amount of fluid to give to patients. We are testing two approaches – one with less fluid and earlier use of medication, where required, is being compared to the conventional treatment where a larger amount of fluid is given during the first few hours of treatment. We are particularly interested in comparing the effects on some markers of inflammation measured on blood samples taken from participants during their treatment.

Patients who take part in the trial will be **randomised** (like tossing a coin) to one or other treatment arms. Neither the patient nor their doctor can control which treatment is allocated.

Because it is important to treat your low blood pressure quickly, we would like your **provisional non-binding** agreement to enrol you in the study. Once treatment is underway we will provide you with more detailed information about the trial and give you the opportunity to ask any questions.

If you are happy to continue in the trial you will be asked to sign a consent form. If, after having read the information, you decide you do not wish to proceed then we will withdraw you from the study and your treatment will proceed as normal under the guidance of your doctor.

You are under no obligation to take part in this research. Choosing not to take part, or providing initial agreement and subsequently withdrawing, will have no adverse impact on the care you receive or the relationship between you and the treating team.

If you do decide to assist with this research you will be helping us to answer an important question that may lead to improvements in care for patients in the future.
